# A peer group intervention implemented by community volunteers increased HIV prevention knowledge

**DOI:** 10.1186/s12889-022-14715-3

**Published:** 2023-02-10

**Authors:** Lily C. Kumbani, Diana L. Jere, Chimwemwe K. Banda, Cecilia Chang, Li Liu, Linda L. McCreary, Crystal L. Patil, Kathleen F. Norr

**Affiliations:** 1 Kamuzu University of Health Sciences , Private Bag 360, Blantyre, Malawi; 2 School of Public Health, University of Illinois Chicago, 1603 W. Taylor Street (M/C 932), Chicago, IL 60612 USA; 3 College of Nursing, University of Illinois Chicago, 845 S. Damen Ave (M/C 806), Chicago, IL 60612 USA

**Keywords:** Community implementation, HIV prevention knowledge, Peer group intervention, Rural health, Malawi

## Abstract

**Background:**

HIV prevention knowledge levels are low in sub-Saharan Africa. In our efficacy study, the *Mzake ndi Mzake* (Friend-to-Friend; hereafter *Mzake*) 6-session peer group intervention, delivered by health workers, improved HIV prevention knowledge and other outcomes in Malawi. To expand HIV prevention approaches, this implementation study tested whether the intervention remained effective when implemented by trained community volunteers. HIV prevention knowledge findings are presented.

**Methods:**

Using a stepped wedge design, three communities implemented the *Mzake* program sequentially in randomly assigned order. Repeated surveys assessed outcomes, and participants served as controls until they completed the program. At Time 2, Community 1 became the intervention group, and at Time 3, Communities 1 and 2 were the intervention group. HIV prevention knowledge, the primary outcome, was assessed through two indicators: UNAIDS comprehensive knowledge (UNAIDS Knowledge), defined as correctly answering five HIV prevention questions (Yes/No), and a 9-item HIV/PMTCT Knowledge Index (number correct). Multivariate generalized estimating equation logistic regression (UNAIDS Knowledge) and mixed-effects regression models (HIV/PMTCT Knowledge Index) were used to assess knowledge controlling for five sociodemographic factors.

**Results:**

In bivariate analyses of UNAIDS Knowledge, more persons answered correctly in the intervention group than the control group at Time 2 (56.8% vs. 47.9%, *p*  < 0.01), but the difference was not significant at Time 3. In logistic regression, there was a significant linear increase in the proportion who correctly answered all questions in the control group, but the increase was significantly higher in the intervention group (log-odds estimate = 0.17, SE = 0.06, *p*-value < 0.01). The HIV/PMTCT Knowledge Index scores increased over time for both groups, but in the intervention group the increase was significantly higher than the control group (0.11 at Time 2; 0.21 at Time 3). In youth and adult subsamples analyses, the intervention was highly effective in increasing knowledge for youth, but not for adults.

**Conclusion:**

This implementation study showed that *Mzake* was effective in increasing HIV prevention knowledge when delivered by community members. Community approaches offer an important strategy to increase HIV prevention in rural communities without burdening healthcare systems.

**Trial registration:**

ClinicalTrials.gov NCT02765659. Registered 06/05/2016

**Supplementary Information:**

The online version contains supplementary material available at 10.1186/s12889-022-14715-3.

## Background

Ending new HIV infections is a global priority, especially in sub-Saharan Africa [1]. The importance of HIV prevention knowledge was recognized early in the pandemic as a critical component of HIV prevention [2]. A sound understanding of how HIV is and is not transmitted allows individuals to identify which behaviors will protect against transmission and is associated with less stigmatization of persons living with HIV, which can positively influence HIV testing and treatment [2–6].

Despite the importance of knowledge about HIV prevention, knowledge levels remain low throughout sub-Saharan Africa [7]. The most widely used measure of HIV prevention knowledge is a set of five questions about sexual transmission and common misconceptions developed by the Joint United Nations Program on HIV/AIDS (UNAIDS) [8, 9]. UNAIDS defines knowledge as comprehensive if all five questions are answered correctly. A recent meta-analysis of data from 33 African countries between 2003 and 2015 reported an average relative UNAIDS comprehensive knowledge increase of only 1% per year [7]. In Malawi, comprehensive knowledge increased substantially for both men and women between 2004 and 2010, but there has been very little change between 2010 and 2016 [10]. Today fewer than half of Malawian adults ages 15–45 have comprehensive knowledge. In the most recent Malawi Demographic and Health Survey (MDHS) [11], only 42% of women and 48% of men had comprehensive HIV knowledge. Among youth ages 15–19, comprehensive knowledge was even lower, only 39% for young women and 43% for young men.

Although comprehensive HIV knowledge is low in Malawi and other African countries, there is evidence that interventions can increase HIV prevention knowledge. Systematic reviews and meta-analyses of the effect of HIV educational interventions in sub-Saharan Africa established that these interventions increased knowledge, and that peer-based and theoretically-grounded interventions were particularly effective in increasing HIV knowledge [6, 12–16]. Several studies in these reviews also showed that increased knowledge was related to risk reduction behaviors such as increased condom use. However, there are long delays between development of evidence-based interventions and their dissemination, and this has been widely noted throughout public health including HIV prevention programs in Africa [17].

To address this gap between intervention development and uptake, our research team conducted a hybrid effectiveness-implementation study testing whether community volunteers could implement an evidence-based peer group intervention for HIV prevention [18]. The culturally-tailored intervention that we tested, called *Mzake ndi Mzake* (Friend-to-Friend, hereafter *Mzake*), incorporates Bandura’s social-cognitive learning theory [19] to build self-efficacy for behavior change and uses a peer group intervention delivery strategy. Both of these intervention characteristics have been linked with higher intervention efficacy [6, 12, 13, 15, 16]. In our previous efficacy study, this 6-session peer group intervention was implemented by health workers and improved HIV prevention knowledge and other HIV-related behavioral outcomes in rural Malawi [20, 21].

We decided to test whether trained community volunteers could implement the *Mzake* program. This effectiveness-implementation study focused on the community members’ implementation success and whether *Mzake* remained effective in changing knowledge and other HIV prevention outcomes. We also made two modifications for community implementation. The first modification accommodated the persistent health worker shortages in Malawi [22] by changing the peer group facilitators from trained health workers to trained community members. To help community members take ownership of implementation, the second modification added the use of a simple 3-Step Implementation Model (Prepare, Rollout, and Sustain) previously used in South Africa [23, 24].

The purpose of this report is to determine whether one of our primary effectiveness variables, HIV knowledge, increased when this peer group HIV prevention program was implemented by community volunteers in rural Malawi. Community-wide implementation of an efficacious intervention has high potential to change sexual risk and HIV testing behaviors, and thus reduce new HIV infections, without imposing new burdens on the health system.

## Methods

### Design

**Fig. 1:**
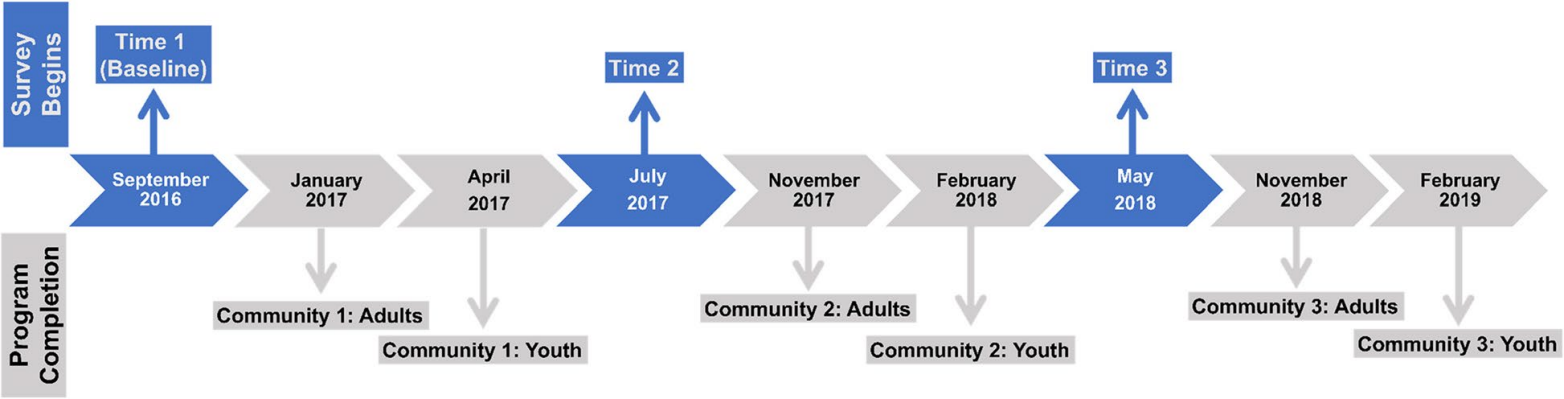
Timeline showing when surveys occurred and completion of the *Mzake* program for adults and youth in each community

We used a hybrid design to equally assess both effectiveness and implementation success and processes in a single study [18]. We also used a stepped wedge design, so that three communities started implementing *Mzake* sequentially in a randomly assigned order [25, 26]. This design is efficient because the repeated measures allow participants to serve as controls until the program is introduced in their community (see Fig. 1). Community volunteers implemented *Mzake* using the 3-step Community Implementation Model: Prepare, Rollout (when each community implemented *Mzake*’s first set of 6-session peer groups for both adults and youth) and Sustain. Effectiveness was evaluated using repeated surveys with the participants in the first set of *Mzake* peer groups. We present results from 3 surveys (Baseline, Time 2, and Time 3). At Baseline, none of the communities had participated in *Mzake*. At Time 2, participants in the first community had completed the program, and participants from the other two communities served as controls. At Time 3, participants in the first and second communities had completed the program, and participants from the third community served as controls. The time between program completion and the first post-program survey was at least 5 months for adults and 3 months for youth for Communities 1 and 2. For Community 1 participants, the time between program completion and the Time 3 survey was at least 15 months for adults and 13 months for youth. Additional details can be found in the study protocol publication [18].

### Setting and preliminary community collaboration

The study took place in Phalombe, a rural district in southern Malawi. The need for HIV prevention is especially high in the Southern Region, where the HIV prevalence is 16%, twice that of other regions in Malawi [27]. Guided by principles of community-based participatory research (CBPR) [28, 29], we met with local leaders and community members, described the intervention, and discussed their willingness to take ownership of the *Mzake* program. All were enthusiastic about introducing HIV prevention in their communities. Community leaders selected the three communities to participate in the study and then determined their randomized implementation order by drawing community names from a basket. In each selected community the leaders identified 10–12 respected adults active in community affairs to serve as volunteers on the Community Coordinating Committee and take charge of organizing implementation.

### Sample size and power calculations

Full details for our power analysis are described in Jere et al. [18]. In brief, sample size calculations for the longitudinal step-wedge design study were carried out using simulations for the two knowledge outcomes, proportions who correctly answered the UNAIDS questions and the mean HIV/PMTCT Knowledge Index. Considering an attrition rate of 25%, a total sample size of 432 individuals per age group was proposed to ensure 80% statistical power for the detection of desired effect sizes at the end of the study. Sample size was determined to be 144 adult and 144 youth at baseline per community (12 adult and 12 youth groups of 12 participants per group). We later learned from leaders that retention of youth might be more problematic than planned for our power analysis. Therefore, we took advantage of having additional facilitators who successfully completed training, allowing us to offer additional groups and increase the youth sample size by 48 per community. A total of 1008 individuals, 460 adults and 548 youth, provided baseline data. At Times 2 and 3, 926 (91.9%) and 923 (91.6%) of the entire baseline sample provided data, respectively.

### Recruitment procedures for the *Mzake* program and its effectiveness evaluation

Using the stepped wedge design, the people who enrolled in the initial sets of peer groups when their community began implementation also comprised the effectiveness evaluation sample. Therefore, these participants had to agree to both complete the 6-session *Mzake* program and complete the repeated evaluation surveys. Other eligibility criteria included: residing in that community, meeting the age range for either an adult group (20 or older) or youth group (13–19), and willing to consent or provide parent consent if under age 18, defined as minors in Malawi law.

Participants in the program were self-selected volunteers who met the recruitment criteria. To recruit community members to participate in *Mzake* and the repeated surveys, that community’s Coordinating Committee held several community meetings in the different locations of the community where peer groups would meet. At each meeting, the Coordinating Committee discussed the *Mzake* program and the effectiveness evaluation. Members of the research team answered any questions and conducted the informed consent process. Interested community members completed the informed consent, signed up for a specific peer group series, and completed the baseline survey. Youth (ages 13–19) and adults (age ≥ 20) were recruited separately because the intervention was conducted in developmentally appropriate ways for youth and adults. The youth recruitment announcement asked those who were under age 18 to bring a parent with them so that they could complete the informed consent process together, including both parental permission and youth assent.

### Intervention:* Mzake *and the 3-step community implementation model

The *Mzake* peer group intervention was developed based upon a conceptual model that integrates health worker-community collaboration, Bandura’s social-cognitive learning theory, and cultural tailoring. There are six sessions, each lasting about 2 h, followed by a celebration with neighbors that includes participant presentations sharing what they have learned and receipt of certificates. Sessions cover HIV transmission and prevention facts, basic reproduction and sexuality information, prevention and treatment of other sexually transmitted infections, testing and treatment as prevention, partner communications, correct condom use, and engaging in community-wide HIV prevention. Every session is built around interactive learning activities including games, discussions, and rehearsal with corrective feedback (role plays). The same group of 10–12 participants and the same two trained co-facilitators meet for all the sessions to build relationships and social support. A group of no more than 12 members allows full participation and is commonly used for nearly all peer group behavior change interventions [6, 12–16].

To respect cultural norms that discourage open discussion of sexuality between youth and adults or males and females, groups are homogenous for gender and age group (adult or youth). The age grouping for youth (13–19) was suggested by community members in formative evaluation for this study. This reflects community perspectives about what age group were likely to share common concerns related to HIV prevention and could discuss these issues comfortably in a single-gender group. Groups of 13–19-year-olds generated good discussion in the efficacy study. Because talking about sexuality with youth is a sensitive topic in Malawi, the peer group facilitators obtained experience and confidence facilitating adult groups before beginning *Mzake* with youth. The *Mzake* program used the same peer group intervention and facilitator training strategies as those used in the original efficacy trial.

In this implementation study, the role of the health workers was modified from the efficacy study. The local health system supported the project by providing health workers who served as volunteer trainers of the community peer group facilitators and provided ongoing support to the project. Before community implementation began, the research team trained healthcare workers who were then able to provide locally available assistance in training new community peer group facilitators. Their training was identical to the training for new community peer group facilitators. Healthcare workers also gained additional experience prior to training peer group leaders because they led the sessions for a pilot of the revised adult manual in a different community. In the first community, the initial training of the peer group leaders was done collaboratively by the research team and the trained health care workers. Later the research team decreased their involvement, and the training of new peer group leaders was conducted by the healthcare workers and experienced community peer group facilitators. By the end of the project several experienced community facilitators became trainers themselves, so that the communities could sustain the project independently.

In this community implementation research, the 3-Step Community Implementation model was used by community volunteers to guide implementation procedures. Each community began implementation in the assigned order and followed the same steps. The Coordinating Committee was appointed by community leaders before implementation began.

During the Prepare step (2–4 months), community volunteers were trained and made an implementation plan. First, the Coordinating Committee members were oriented to the 3-Step Implementation Model and the *Mzake* program in a 3-week training. At the end of training, they developed a detailed community implementation plan for the initial rollout of the Program. Next, they invited 16 community members to serve as volunteer peer group facilitators. The Committee selected potential facilitators based on their literacy in the local language, a history of reliability and active engagement in community affairs, and recognition as a positive role model for HIV-related behaviors. Facilitators’ educational level was substantially higher than the community; all had completed primary school, and most (72%) had attended 2–4 years of secondary school. The 2-week facilitator training was highly interactive with extensive practice and feedback. Although content was fully covered, there was a strong focus on gaining group facilitation skills.

Peer group facilitator training included 16 facilitators in each community to allow for attrition and end up with at least 12 trained facilitators in each community. We planned that each pair of co-facilitators would conduct 2 adult and 2 youth groups, and each group would include 12 community members. This would achieve the sample size needed according to our power calculations. However, in each community all 16 trained facilitators successfully completed training and wanted to offer the program, so we increased the number of peer groups in each community from 12 to 16. This gave us an opportunity to ask the additional 48 youth group members to participate in both *Mzake* and the repeated surveys to allow for possible greater attrition from the youth groups. Additional adults were offered the opportunity to participate in *Mzake* but were not asked to participate in the effectiveness evaluation.

During the Rollout step (3–4 months) in each community, the planned initial set of Mzake peer groups for adults and youth were conducted. The Coordinating Committee worked with the newly trained facilitators to establish places and times for the group session meetings and informed the previously enrolled *Mzake* participants of these logistics. Then the facilitators offered the program. In each community eight pairs of co-facilitators led four groups (two adult and two youth) for a total of 16 adult and 16 youth groups. Participants in each group completed all six sessions plus the celebration. Only four persons who signed up to be in the group failed to complete all six sessions.

After a community completed the Rollout step, that community began the Sustain step which lasted through the end of the grant funding. Committee members and facilitators continued to offer *Mzake* but tailored their plans to meet their own community needs. Communities that started implementation earlier had a longer time for sustaining the program.

### Measures

The outcome of interest in this analysis is HIV prevention knowledge, which we measured using two indicators. UNAIDS comprehensive HIV prevention knowledge (UNAIDS Knowledge) is defined as answering all of 5 knowledge items correctly (Yes/No): if a healthy-looking person can have HIV, whether condom use and having only one uninfected partner reduce the likelihood of getting HIV transmission, and two of the most common local misconceptions about HIV transmission (casual contact and mosquito bites) [9]. This indicator is easily measured and widely used globally, facilitating comparisons across time and countries. However, the measure is not inclusive of all aspects of HIV prevention.

Because prevention of mother to child transmission (PMTCT) is a major focus of HIV prevention, we created a more inclusive measure of HIV prevention knowledge by combining the five UNAIDS Knowledge questions with four additional PMTCT items taken from the MDHS survey [11]. The PMTCT questions asked If HIV can be transmitted during pregnancy, during delivery, and breastfeeding and whether drugs can reduce this risk. The HIV/PMTCT Knowledge Index is a continuous variable (score = number of items correct, range = 0–9).

Sociodemographic factors controlled for in multivariate longitudinal analyses included the baseline variables of sex (self-identified), age group (youth or adult), education, and level of religious involvement in religious activities (very involved versus somewhat or less involved), and the time-varying covariate of whether married or living with partner (yes or no). When examining youth and adults separately, baseline age was included as a covariate, and whether currently in school (yes, no) was added as a time-varying covariate for youth sample.

### Statistical analyses

Data summaries and analyses were performed for the total sample and then for the youth and adult subsamples separately. Time 2 and 3 proportions of UNAIDS knowledge were compared to baseline proportions using the McNemar’s test for paired binary data. Paired t-tests were employed to compare the mean HIV/PMTCT Knowledge Index at Times 2 and 3 to that of the baseline. To evaluate bivariate relationships between sociodemographic factors, which were all categorical variables, and the dichotomous UNAIDS Knowledge outcome at each time point, Chi-squared tests were employed. The relationships between sociodemographic factors and the HIV/PMTCT Knowledge Index were evaluated using t-tests for binary factors or analysis of variance models (ANOVA) for factors with more than two levels.

In the multivariate analyses, intervention effectiveness was identified when the intervention group demonstrated significantly higher increasing trend in knowledge, and there were significant intervention-control differences at each of the follow-up time points, either in odds ratio for the binary UNAIDS Knowledge, or in mean differences for the HIV/PMTCT Knowledge Index. The magnitudes of the intervention effects over time were estimated using multivariate models for longitudinal design, controlling for baseline and time-varying sociodemographic factors. Specifically, generalized estimating equation (GEE) models were used to model the dichotomous UNAIDS Knowledge over time. Mixed-effects regression models (MRM) were used to model the repeatedly measured HIV/PMTCT Knowledge Index. Variance-covariance structures for repeated measures (in GEE) and random effects plus error structure (in MRM) were selected using fit statistics such as Akaike information criterion (AIC) and likelihood ratio tests when possible. Due to the stepped wedge design, in which all individuals were in the control group at baseline, group effect was not entered in the regression models as an additive fixed effect. Instead, group by time interaction terms, which indicated group differences in over-time trends, were the primary focus of inferences. For the total sample, significant fixed-effects sociodemographic factors, (baseline age group, self-identified sex,, education level, religious involvement) and the time-varying effect (partner status) were controlled after backward model selections in the longitudinal models. For the youth and adult subsample analyses, baseline age replaced age group and whether currently in school was added as a time-varying covariate for the Youth Subsample only. Covariates-adjusted time-point specific group differences and their 95% confidence interval estimates were reported for both knowledge measures (group odds ratios, ORs, for UNAIDS Knowledge and group mean differences for HIV/PMTCT Knowledge index). All statistical tests were 2-tailed, controlling for Type I error probability of 0.05. The statistical software program SAS 9.4 [30] was used for all statistical analyses.

### Ethical considerations

All stages of this study were conducted in accordance with the principles of the Helsinki Declaration. This study received ethical approval from the University of Illinois at Chicago and the Malawi College of Medicine Research Committee before study recruitment began. After the community meeting introducing the study, those who were interested in participating completed individual signed informed consent. Adults and youth over age 17 consented as adults. Youth ages 13–17 are minors under Malawi law. A waiver was obtained to allow obtaining permission from only one parent or guardian. Because youth were old enough to understand the consent process, each youth and one parent or guardian completed the consent process together. The study was explained to them together and questions from both parent and youth were answered. Then both parent and youth signed the consent form. To minimize any perceived parental coercion, youth assent was reconfirmed verbally prior to the first data collection when the parent was not present.

## Results

### Participant characteristics

A total of 1008 community members were recruited to participate. There were 338 participants from Community 1 and 335 each from Communities 2 and 3. At baseline, just over half (51%) were female, and 54.4% were youth. Nearly half (48.9%) did not complete primary school, 36.7% completed primary school, and 14.4% had completed secondary school. When asked about level of involvement in religious activities, 64.5% reported being very involved. At Baseline, 43% of participants were married or living with a partner; this increased to 50% at Time 3.

### HIV and PMCTC Knowledge outcomes: bivariate relationships

Bivariate relationships between sociodemographic factors and study condition (intervention vs. control) with each of the two outcomes, correctly answering all UNAIDS Knowledge questions (Yes/No) and scores on the HIV/PMTCT Knowledge Index, are in Table 1. Adults had better knowledge than youth across all three timepoints. These age-group differences were statistically significant at baseline and Time 2 for UNAIDS Knowledge. Mean scores on the HIV/PMTCT Knowledge Index were significantly higher in adults at all three time points. There was no statistical difference for either knowledge indicator for males or females. Higher education level was significantly associated with better UNAIDS knowledge at all three time points. Those who had high religious involvement had significantly higher mean HIV/PMTCT Knowledge Index score at Time 2, but not at baseline or Time 3.

**Table 1:** Bivariate analysis of UNAIDS Knowledge (%, all correct) and mean HIV/PMTCT Knowledge Index at each time point

	**Baseline**			**Time 2**			**Time 3**		
	**N**	**UNAIDS** **n (%)**	**HIV/PMTCT Knowledge Index** **Mean (SD)**	**N**	**UNAIDS** **n (%)**	**HIV/PMTCT Knowledge Index** **Mean (SD)**	**N**	**UNAIDS** **n (%)**	**HIV/PMTCT Knowledge Index** **Mean (SD)**
**Total sample**	1008	429 (42.56)	7.44 (1.60)	926	471 (50.86)	7.76 (1.50)	923	521 (56.45)	7.98 (1.36)
**Age Group**									
Youth	548	213 (38.87)**	7.16 (1.73)***	500	233 (46.60)**	7.57 (1.65)***	489	273 (55.83)	7.91 (1.44)*
Adult	460	216 (46.96)	7.78 (1.36)	426	238 (55.87)	8.00 (1.26)	434	249 (57.37)	8.09 (1.24)
**Sex**									
Male	494	218 (44.13)	7.49 (1.58)	445	234 (52.58)	7.82 (1.41)	448	256 (57.14)	8.07 (1.26)
Female	514	211 (41.05)	7.40 (1.62)	481	237 (49.27)	7.72 (1.58)	475	265 (55.79)	7.92 (1.43)
**Education**									
Did not complete primary school	493	167 (33.87)	7.00 (1.80)***	459	189 (41.18)***	7.35 (1.77)***	465	240 (51.61)**	7.72Z (1.56)***
Complete primary school	370	180 (48.65)	7.79 (1.33)	344	201 (58.43)	8.14 (1.01)	340	201 (59.12)	8.20 (1.12)
Complete secondary school	145	82 (56.55)	8.10 (0.97)	123	81 (65.85)	8.28 (1.07)	118	80 (67.80)	8.47 (0.74)
**Community**									
Community 1	338	154 (45.56)	7.47 (1.75)	299	171 (57.19)*	7.99 (1.30)*	305	176 (57.70)	8.10 (1.30)
Community 2	335	132 (39.40)	7.34 (1.60)	319	148 (46.39)	7.61 (1.62)	310	186 (60.00)	7.99 (1.40)
Community 3	335	143 (42.69)	7.52 (1.43)	308	152 (49.35)	7.71 (1.53)	308	160 (51.95)	7.89 (1.35)
**Religious Involvement**									
Less involved	358	141 (39.39)	7.29 (1.72)*	330	169 (51.21)	7.68 (1.58)	329	190 (57.75)	7.90 (1.50)
Very involved	650	288 (44.31)	7.53 (1.53)	596	302 (50.67)	7.82 (1.45)	594	331 (55.72)	8.04 (1.26)
**Partner Status Baseline**									
Single	572	226 (39.51)*	7.21 (1.74)***	-	-	-	-	-	-
Married or living with partner	436	203 (46.56)	7.75 (1.34)	-	-	-	-	-	-
**Partner Status Time 2**									
Single	-	-	-	535	244 (45.61)***	7.54 (1.70)***	-	-	-
Married or living with partner	-	-	-	395	228 (57.72)	8.06 (1.13)	-	-	-
**Partner Status Time 3**									
Single	-	-	-	-	-	-	460	253 (55.00)	7.91 (1.50)
Married or living with partner	-	-	-	-	-	-	466	268 (57.51)	8.06 (1.20)
**Intervention Condition Baseline**									
Control	1008	429 (42.56)	7.44 (1.60)	-	-	-	-	-	-
**Intervention Condition Time 2**									
Control	-	-	-	627	300 (47.85)*	7.66 (1.58)**	-	-	-
Intervention	-	-	-	303	172 (56.77)	7.97 (1.32)	-	-	-
**Intervention Condition Time 3**									
Control	-	-	-	-	-	-	308	160 (51.95)	7.89 (1.35)
Intervention	-	-	-	-	-	-	619	362 (58.48)	8.03 (1.37)

Significance level: * < .05, ** < .01, *** < .001

At Time 2, when participants from Community 1 had completed the *Mzake* program, the proportion of those who correctly answered UNAIDS questions and the mean knowledge score in Community 1 were significantly higher than the other two communities who were serving as controls. No other statistical differences were identified between communities in the bivariate analyses. People who were married or living with a partner were more likely to get all five UNAIDS Knowledge questions correct and had higher mean HIV/PMTCT Knowledge Index scores than those who identified as single at baseline and Time 2.

**Fig. 2:**
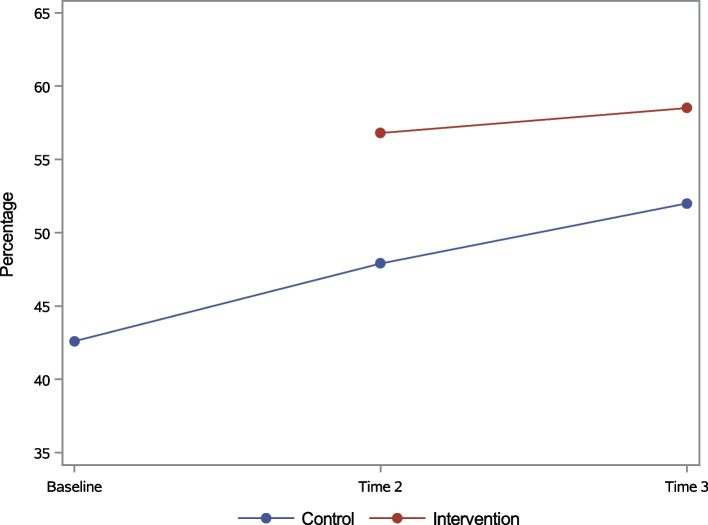
UNAIDS Knowledge (%, all correct) over time for the total sample

**Fig. 3:**
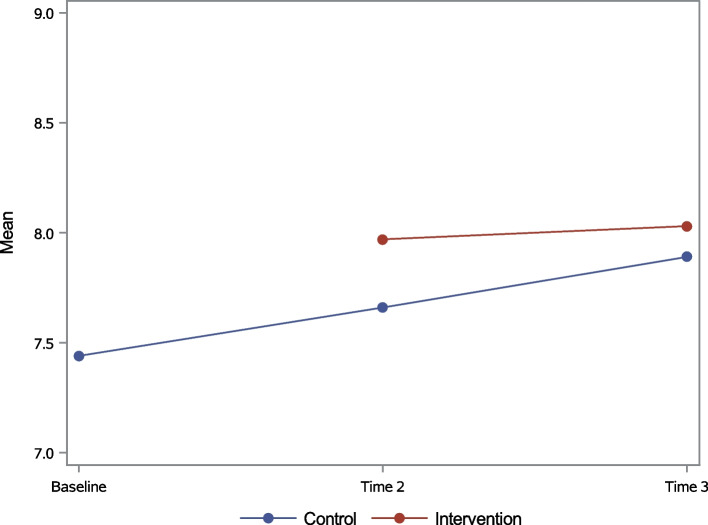
HIV/PMTCT Knowledge Index (mean) for the total sample

In general, both HIV knowledge indicators improved over time. The proportion of individuals who correctly answered all UNAIDS Knowledge questions increased from 43% at baseline to 51% at Time 2, and 56% at Time 3. The mean HIV/PMTCT Knowledge Index score increased from 7.44 at baseline to 7.76 at Time 2, and to 7.89 points at Time 3. In bivariate analyses, the intervention-control differences were the largest and statistically significant at Time 2 for both UNAIDS Knowledge (56.8% versus 47.9%, Fig. 2) and mean HIV/PMTCT Knowledge Index scores (7.97 versus 7.66, Fig. 3). Significance did not hold at Time 3 for either knowledge variable.

### Change in UNAIDS Knowledge and HIV/PMCTC Knowledge Index: multivariate predictors

In the final multivariate GEE logistic regression models for the binary UNAIDS Knowledge (Table 2), there was a significantly positive linear increase in the proportions of those who correctly answered all five questions in the control group (Table 2, time effect log-odds estimate = 0.18, SE = 0.06, *p*-value <0.001). The linear increase in the intervention group was significantly higher, as indicated by a significantly positive time-by-intervention interaction (log-odds estimate = 0.17, SE = 0.06, *p*-value < 0.01).

**Table 2:** Results of multivariate GEE model for UNAIDS Knowledge (Range 0 - 1) for the total sample

	**Estimate (SE)**	***P*-value**	**OR (95% CI)**
**Education**			
Did not complete Primary School	ref	ref	ref
Complete primary school	0.53 (0.11)	< 0.0001	1.69 (1.37, 2.09)
Complete secondary school	0.88 (0.15)	< 0.0001	2.41 (1.79, 3.26)
**Age Group**			
Adult	0.19 (0.10)	0.06	1.21 (0.99, 1.47)
**Time**	0.18 (0.06)	0.001	-
Time * Intervention	0.17 (0.06)	0.01	-
**OR (95% CI), intervention-control difference at Time 2**			1.39 (1.10, 1.77)
**OR (95% CI), intervention-control difference at Time 3**			1.64 (1.15, 2.35)

Education level was significantly associated with answering all five UNAIDS Knowledge questions correctly. Those who completed primary school at baseline were 1.69 times more likely to correctly answer all questions than those who did not complete primary school (*p*-value < 0.0001); those who completed secondary school were 2.41 times more likely to correctly answer the UNAIDS questions than those who did not complete primary school *(p*-value < 0001).

Fixed-effects estimates from the MRM models for the continuous HIV/PMTCT Knowledge Index are in Table 3. Model selections for random effects in the MRM revealed significant individual deviations in both the baseline knowledge and linear trends. The intra-class correlation coefficient (ICC) from a random intercept model was 0.65, indicating a moderate correlation between repeatedly measured knowledge scores. Although the linear increase in the control group was significant (estimate = 0.20 point per year, *p*-value < 0.0001), the increase in the intervention group was significantly higher than the control group by 0.11 point per year (*p*-value = 0.003). Therefore, on a 9-point scale, the mean knowledge score in the intervention group was estimated at 0.11 points higher than control at Time 2, and 0.21 points higher at Time 3 (both were statistically significant). Other significant factors that were associated with improvement in the HIV/PMTCT Knowledge Index were being adult rather than youth, being married or living with a partner rather than being single and having completed primary school or secondary school.

**Table 3:** MRM model results for HIV/PMTCT Knowledge Index (Range 0-9) for total sample

	**Estimate (SE)**	***P*-value**
**Education:**		
Did not complete Primary School	ref	ref
Complete primary school	0.64 (0.08)	< 0.0001
Complete secondary school	0.86 (0.11)	< 0.0001
**Age Group:**		
Adult	0.19 (0.09)	0.03
**Partner Status:**		
Married or living with a partner	0.15 (0.06)	0.01
**Time**	0.20 (0.03)	< 0.0001
Time * Intervention	0.11 (0.04)	0.003
**Intervention-control difference at Time 2 (95% CI)**	0.11 (0.04, 0.18)	
**Intervention-control difference at Time 3 (95% CI)**	0.21 (0.07, 0.35)	
**ICC**	0.65	

### Knowledge outcomes: youth and adults

Next, we examined the impact of the intervention on UNAIDS Knowledge and the HIV/PMCTC Knowledge Index for youth and adults separately. Youth and Adult subsample descriptions and bivariate tables are in Additional files 1 and 2, respectively. The smaller sample sizes and reduced statistical power resulted in fewer significant differences within the age sub-samples. The effects of education and religious involvement on both knowledge indicators remained consistent in the subsample bivariate results. However, the effect of gender worked differently in the separate samples. Male youth had significantly higher mean scores compared to female youth (7.4 versus 6.9 at T1, 7.8 versus 7.3 at T2, and 8.1 versus 7.7 at T3, α = 0.05 level). Among the adults, female adults had higher mean knowledge scores than male adults at T1 and T2 (8.0 versus 7.6 at T1, 8.2 versus 7.8 at T2, both significant). The gender difference at T3 was not significant. In addition, those adults who were married or living with partner had significantly higher knowledge means (about 0.4 point higher), while such partner status effect was not found in the bivariate analysis in the youth sample.

**Fig. 4:**
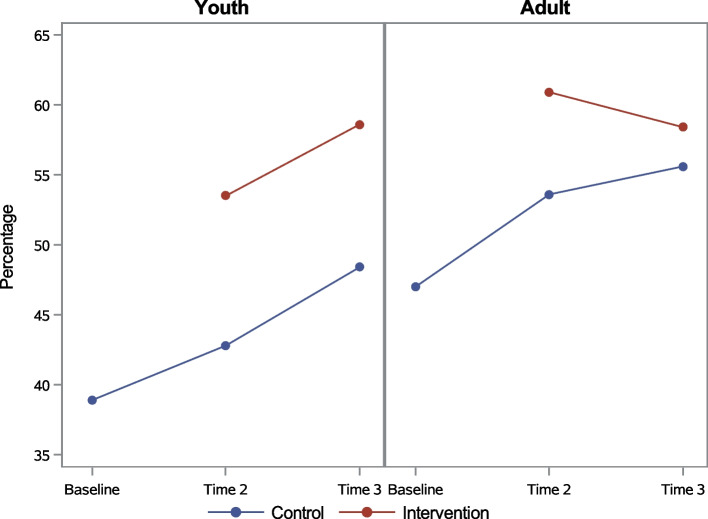
UNAIDS Knowledge by age group

**Fig. 5:**
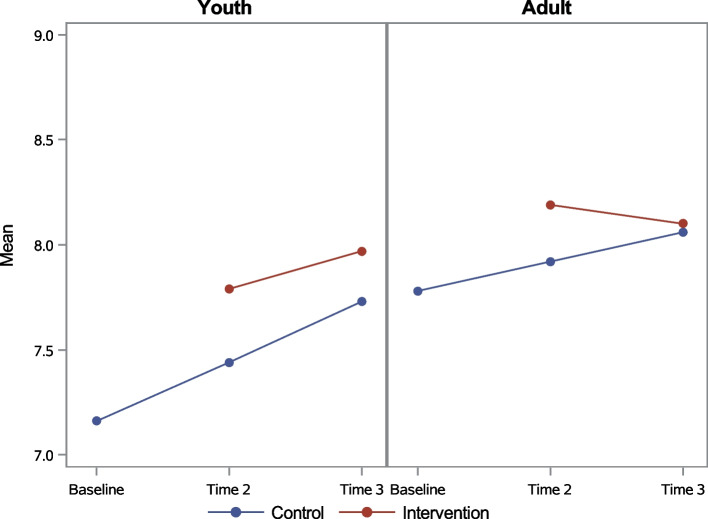
HIV/PMTCT Knowledge Index (mean) by age group

In general, adult baseline knowledge levels were higher than youths, and knowledge increased over time for both adults and youth regardless of intervention group (Figs. 4 and 5). Among youth, Chi-squared tests showed that the intervention group did significantly better in UNAIDS Knowledge questions at both T2 (53.5% versus 42.8%) and T3 (58.6% versus 48.4%), while t-tests showed significant advantage in intervention group mean score on the HIV/PMTCT Knowledge Index at T2 only (7.8 versus 7.4). Among adults, a significant intervention-control group difference was only found at Time 2, when the mean score on the HIV/PMTCT Knowledge Index was 8.2 for adults who completed the intervention and 7.9 for adults in the control group.

Multivariate GEE model results for the youth and adult separate analyses on UNAIDS Knowledge are in Table 4. Among youth, the control group had a significantly positive linear trend in logit (i.e., log odds of the probability of correctly answering the UNAIDS questions) with estimate size of 0.21 (*p*-value < 0.01). The difference in the trend in the logit regression for the intervention group is of the same size (estimate = 0.21, *p*-value < 0.01), indicating that the speed of increase in the intervention group doubled over time. The OR for intervention effects were also significant for youth at both time points 2 (OR = 1.23, CI = 1.04–1.46) and 3 (OR = 1.52, 95% CI = 1.09–2.12). Such intervention effects (significant intervention-by-time interaction and time-point specific group effects) were not found in the adult sample.

**Table 4:** Results of GEE model for UNAIDS Knowledge (Range 0 - 1) for youth and adults

	**Youth *N* = 548**			**Adult *N* = 460**		
	**Estimate (SE)**	***p*-value**	**OR (95% CI)**	**Estimate (SE)**	***p*-value**	**OR (95% CI)**
**Education**						
Did not complete Primary School	ref	ref	ref	ref	ref	ref
Complete primary school	0.66 (0.14)	< 0.0001	1.94 (1.47, 2.56)	.31 (0.17)	0.06	1.36 (0.98, 1.88)
Complete secondary school	0.81 (0.24)	0.001	2.24 (1.40, 3.59)	.86 (0.20)	< 0.0001	2.37 (1.60, 3.52)
**Religious Involvement**						
Very Involved	-0.31 (0.14)	0.02	0.73 (0.56, 0.96)	0.36 (0.16)	0.02	1.43 (1.05, 1.95)
**Time**	0.21 (0.08)	0.01	-	0.15 (0.08)	0.06	-
Time * Intervention	0.21 (0.08)	0.01	-	0.11 (0.09)	0.20	-
**OR (95% CI), intervention-control difference at Time 2**			1.23 (1.04, 1.46)			1.12 (0.94, 1.33)
**OR (95% CI), intervention-control difference at Time 3**			1.52 (1.09, 2.12)			1.26 0(0.89, 1.78)

Similarly, intervention effectiveness was identified for the HIV/PMTCT knowledge index (Table 5) in the youth sample, but not in the adult sample. Specifically, the yearly increase in the mean HIV/PMTCT Knowledge Index score among youth control participants was 0.31 unit in the 0–9 knowledge scale (*p*-value <0.0001). The youth in the intervention group had an additional yearly increase of 0.12 unit (*p*-value = 0.02), The Time 2 (0.12 unit) and Time 3 (0.25 unit) group differences in the mean HIV/PMTCT Knowledge Index scores were both statistically significant. In the adult sample, however, the control group yearly increase was significant at 0.10 unit per year, while the rate of increase in the intervention group was not statistically different from the control adults. In addition, T2 and T3 group differences were not significant at α < 0.05 level.

**Table 5:** Results of MRM model for HIV/PMTCT Knowledge Index for youth and adults

	**Youth *N* = 548**		**Adult *N* = 460**	
	**Estimate (SE)**	***p*-value**	**Estimate (SE)**	***p*-value**
**Education**				
Did not complete Primary School	Reference	Reference	Reference	Reference
Complete primary school	0.84 (0.12)	< 0.0001	0.35 (0.11)	0.001
Complete secondary school	0.95 (0.19)	< 0.0001	0.78 (0.13)	< 0.0001
**Sex**				
Female vs. Male	-0.36 (0.11)	0.001	0.41 (0.10)	< 0.0001
**Partner Status**				
Married or living with a partner	-	-	0.25 (0.08)	0.003
**Religious Involvement**				
Very involved	-	-	0.41 (0.10)	< 0.0001
**Time**	0.31 (0.05)	< 0.0001	0.10 (0.04)	0.02
Time * Intervention	0.12 (0.05)	0.02	0.07 (0.05)	0.11
**Intervention-control difference at Time 2 (95% CI)**	0.12 (0.02, 0.23)		0.07 (-0.02, 0.16)	
**Intervention-control difference at Time 3 (95% CI)**	0.25 (0.04, 0.46)		0.15 (-0.03, 0.32)	
**ICC**	0.72		0.65	

When youth and adult samples were examined separately, some sociodemographic factors affected the knowledge measures consistently, while others affected knowledge in opposite ways for youth and adults. Education effects on knowledge were consistent between youth and adult samples; higher education improved both knowledge measures. Conversely, multivariate modeling results confirmed the opposite effects of self-identified sex shown in the bivariate analyses of separate age groups. Female youth had lower mean scores on the HIV/PMTCT Knowledge Index compared to male youth (estimate = -0.36, *p*-value <0.001). Female adults had higher mean scores than male adults (estimate = 0.41, *p*-value < 0.0001). Being very involved in religion (estimate = 0.41, *p*-value < 0.0001) and married or living with a partner (estimate = 0.25, *p*-value = 0.003) improved the HIV/PMTCT Knowledge Index scores in adults but had no effect among the youth.

## Discussion

When *Mzake* was implemented by trained community volunteers in southern rural Malawi, participants’ HIV prevention knowledge increased for both indicators. These results are similar to the initial efficacy study [20, 21]. When we examined youth and adults separately, *Mzake* was effective for youth but not adults. The impact on youth is especially important because they had less HIV knowledge at baseline and are substantially more vulnerable to new HIV infection than adults. These findings confirm that the intervention remained effective in improving prevention knowledge when delivered by community volunteers rather than by health workers. Results also provide further evidence that peer group interventions guided by Bandura’s social-cognitive learning theory [19] are effective in increasing HIV prevention knowledge, confirming findings of previous systematic reviews and meta-analyses [6, 12–17].

Our findings that knowledge is greater for those with more education and for adults rather than youth are consistent with previous findings in both Malawi and other African countries [7, 10, 31]. In this study, being adult was associated with higher knowledge for the total sample. However, in the subsample analyses, age as a continuous variable was not a significant predictor of either knowledge indicator.

In contrast to previous research in Malawi and other African countries [10, 32], gender was not a consistent predictor of HIV knowledge in multivariate analyses for this study. For the total sample, gender was not a predictor of either HIV knowledge indicator. When adults and youth were analyzed separately, gender (measured as self-identified sex) did not predict UNAIDS Knowledge. This lack of a significant difference is consistent with the substantial decreases in the gender gap in comprehensive knowledge in the MDHS from 2004, when 16% more men had comprehensive knowledge than women, to a gap of only 6% in 2015-16 [10, 11]. However, for HIV/PMTCT Knowledge Index scores, gender had a different relationship to knowledge among youth and adults. Among youth, females had lower scores than males; but among adults, females had higher HIV/PMTCT Knowledge Index scores. The lower knowledge among female youth is surprising since all students are exposed to the mandatory Life Skills curriculum. Lower knowledge among young women could reflect gender norms emphasizing that young women should not discuss or show interest in sexuality-related issues [32–35]. The greater HIV/PMTCT Knowledge Index scores among female adults compared to male adults has not been reported previously in Malawi. This finding may reflect the impact of PMTCT programs, which now provide HIV counselling and testing for 95% or more of all pregnant women at their first prenatal visit [36, 37]. Few men accompany their wives to antenatal clinic or participate in couples testing despite clinic policies and government efforts encouraging male participation [38]. Among youth in this study, the majority have not yet become a parent and therefore were not exposed to this knowledge at prenatal clinics. Thus, adult women are more likely to receive information focused on PMTCT than men and younger women.

Religious involvement was another significant factor that affected HIV knowledge differently for youth and adults. For adults, high religious involvement related to higher HIV knowledge for both indicators. For youth, high religious involvement related to lower HIV knowledge for the UNAIDS Knowledge indicator only. In Malawi and most other African countries, faith-based organizations disseminate a great deal of HIV-related information. However, a recent systematic review found that many faith-based organizations in Africa emphasize abstinence-only messages for youth and discourage condom use, which may affect the types of HIV prevention messages they receive [39]. Although Malawi’s 2015–2020 National strategic plan for HIV/AIDS [40] recommends engaging religious leaders to address the HIV prevention, it is important that faith-based messaging is accurate and appropriate for youth as well as adults.

Although the baseline UNAIDS Knowledge levels for this Phalombe sample were close to the national average, there was a significant increase for both knowledge indicators over time in addition to the time by intervention effects. Given that UNAIDS comprehensive HIV knowledge increased very little in Malawi from 2010 to 2016 [11], this overall increase was somewhat surprising. Apart from the *Mzake* program, other factors may have influenced knowledge change over time. Ongoing national HIV prevention efforts on billboards, newspapers, and radio programs, some of which were directed at youth, should affect general HIV knowledge for both youth and adults [41]. Additionally, since 2014, both primary and secondary public schools are required to teach the Life Skills curriculum. HIV content is covered comprehensively and includes basic facts about HIV prevention, testing and treatment; reducing stigma and discrimination; sexual and reproductive health; and sexuality, including sexual identity. Nearly three-quarters of youth in this sample were still attending school. However, delivery of the Life Skills curriculum is not as comprehensive as the content. In addition to poor teaching conditions, some teachers find some of the content uncomfortable for them to share with students [42–44]. The lack of teacher and community support for teaching sexual education to youth is especially problematic in rural schools [43], Because there was strong evidence of diffusion from those receiving *Mzake* to their social networks in the efficacy study [45], potential spillover across communities may also account for some of the increase in knowledge over time. The next MDHS should provide information to help clarify whether the increase in knowledge is nationwide or may be related to these localized indirect effects of *Mzake*.

### Limitations

One limitation of this study is the limited number of validated HIV prevention knowledge measures available. Although widely used and integrated into most national surveys, the UNAIDS measure was developed in 2002 and does not include questions about newer prevention and treatment options. We expanded the UNAIDS Knowledge measure to some extent by adding items about PMTCT. However, our HIV/PMTCT Knowledge Index also suffers from a ceiling effect, with mean scores close to the maximum possible score of 9, especially for the intervention group. Ceiling effects may make identification of the impact of an intervention harder to detect as there is little room for measurable improvement [46]. Since the *Mzake* program was developed, only three new measures with good psychometric properties have been developed appropriate for Africa [47–49]. There is a clear need for a new psychometrically sound knowledge measure inclusive of new aspects of HIV prevention and appropriate for global use.

### Implications

Despite years of mass media campaigns and the introduction of HIV prevention education in schools, HIV prevention knowledge has shown little improvement in Malawi since 2010. This community implementation study found that *Mzake* is effective in increasing HIV prevention knowledge when organized and delivered by community members. Community implementation is an innovative way to increase HIV prevention knowledge in rural communities without burdening the health system.

## Supplementary Information

**Additional file 1.** Youth Subsample: Bivariate relationships between each covariate and knowledge measures (UNAIDS Knowledge and HIV/PMTCT Knowledge Index). Table showing bivariate relationships between all covariates and each knowledge outcome at Baseline, Survey 2 and Survey 3 for the Youth Subsample.

**Additional file 2.** Adult Subsample: Bivariate relationships between each covariate and knowledge measures (UNAIDS Knowledge and HIV/PMTCT Knowledge Index). Table showing bivariate relationships between all covariates and each knowledge outcome at Baseline, Survey 2 and Survey 3 for the Adult Subsample.

## Data Availability

The datasets generated and/or analyzed during the current study are not yet publicly available but will be made available based on reasonable request by emailing the corresponding author.

## Abbreviations

HIV: Human immunodeficiency virus
MDHS: Malawi Demographic and Health Survey
PMTCT: Prevention of mother to child transmission
UNAIDS: Joint United Nations Programme on HIV/AIDS
WHO: World Health Organization

## References

[1] Joint United Nations Programme on HIV/AIDS (2030). On the fast-track to end AIDS by 2030: focus on location and population.

[2] Ndugwa Kabwama S, Berg-Beckhoff G (2015). The association between HIV/AIDS-related knowledge and perception of risk for infection: a systematic review. Perspect Public Health.

[3] Letshwenyo-Maruatona SB, Madisa M, Boitshwarelo T, George-Kefilwe B, Kingori C, Ice G (2019). Association between HIV/AIDS knowledge and stigma towards people living with HIV/AIDS in Botswana. Afr J AIDS Res.

[4] Sano Y, Antabe R, Atuoye KN, Hussey LK, Bayne J, Galaa SZ (2016). Persistent misconceptions about HIV transmission among males and females in Malawi. BMC Int Health Hum Rights.

[5] UNAIDS. Evidence for eliminating HIV-related stigma and discrimination — guidance for countries to implement effective programmes to eliminate HIV-related stigma and discrimination in six setting. UNAIDS report. 2020. https://www.unaids.org/en/resources/documents/2020/eliminating-discrimination-guidance. Accessed 15 Nov 2021.

[6] Faust L, Yaya S (2018). The effect of HIV educational interventions on HIV-related knowledge, condom use, and HIV incidence in sub-Saharan Africa: a systematic review and meta-analysis. BMC Public Health.

[7] Chan BT, Tsai AC (2018). HIV knowledge trends during an era of rapid antiretroviral therapy scale-up: an analysis of 33 sub‐Saharan African countries. J Int AIDS Soc.

[8] Amon J, Brown T, Hogle J, Macneil J, Magnani R, Mills S, et al. Behavioral surveillance surveys-guidelines for repeated behavioral surveys in populations at risk of HIV. 2000. https://www.fhi360.org. Accessed 15 Feb 2021.

[9] Joint United Nations Programme on HIV/AIDS. Monitoring the declaration of commitment on HIV/AIDS: guidelines on construction of core indicators. 2003.

[10] Chirwa GC (2020). “Who knows more, and why?” Explaining socioeconomic-related inequality in knowledge about HIV in Malawi. Sci Afr.

[11] National Statistical Office (NSO) [Malawi] and ICF. Malawi Demographic and Health Survey 2015-16. Zomba, Malawi, and Rockville, USA. NSO and ICF. 2017.

[12] Mwale M, Muula AS (2017). Systematic review: a review of adolescent behavior change interventions [BCI] and their effectiveness in HIV and AIDS prevention in sub-Saharan Africa. BMC Public Health.

[13] Albarracin J, Albarracin D, Durantini M (2008). Effects of HIV-prevention interventions for samples with higher and lower percents of Latinos and Latin Americans: a meta-analysis of change in condom use and knowledge. AIDS Behav.

[14] Medley A, Kennedy C, O’Reilly K, Sweat M (2009). Effectiveness of peer education interventions for HIV prevention in developing countries: a systematic review and meta-analysis. AIDS Educ Prev.

[15] Noar S (2008). Behavioral interventions to reduce HIV-related sexual risk behavior: review and synthesis of meta-analytic evidence. AIDS Behav.

[16] Vergidis P, Falagas M (2009). Meta-analyses on behavioral interventions to reduce the risk of transmission of HIV. Infect Dis Clin N Am.

[17] Green LW, Ottoson JM, García C, Hiatt RA (2009). Diffusion theory and knowledge dissemination, utilization, and integration in public health. Annu Rev Public Health.

[18] Jere DL, Banda CK, Kumbani LC, Liu L, McCreary LL, Park CG, Patil CL, Norr KF (2018). A hybrid design testing a 3-step implementation model for community scale-up of an HIV prevention intervention in rural Malawi: study protocol. BMC Public Health.

[19] Bandura A (1989). Human agency in social cognitive theory. Am Psychol.

[20] Kaponda CPN, Norr KF, Crittenden KS, Norr JL, McCreary LL, Kachingwe SI (2011). Outcomes of an HIV prevention peer group intervention for rural adults in Malawi. Health Educ Behav.

[21] Dancy BL, Jere DL, Kachingwe SI, Kaponda CP, Norr JL, Norr KF (2014). HIV risk reduction intervention for rural adolescents in Malawi. J HIV/AIDS Soc Serv.

[22] Bickton FM, Lillie T (2019). Strengthening human resources for health in resource-limited countries: the case of medic to medic in Malawi. Malawi Med J.

[23] Bergh A-M, Arsalo I, Malan AF, Patrick M, Pattinson RC, Phillips N (2005). Measuring implementation progress in Kangaroo Mother Care. Acta Paediatr.

[24] Pattinson RC, Arsalo I, Bergh AM, Malan AF, Patrick M, Phillips N (2005). Implementation of kangaroo mother care: a randomized trial of two outreach strategies. Acta Paediatr.

[25] Mdege ND, Man M-S, Taylor Nee Brown CA, Torgerson DJ (2011). Systematic review of stepped wedge cluster randomized trials shows that design is particularly used to evaluate interventions during routine implementation. J Clin Epidemiol.

[26] Woertman W, de Hoop E, Moerbeek M, Zuidema SU, Gerritsen DL, Teerenstra S (2013). Stepped wedge designs could reduce the required sample size in cluster randomized trials. J Clin Epidemiol.

[27] PHIA Project. Malawi population-based HIV impact assessment (MPHIA). 2016.

[28] Dworkin SL, Pinto RM, Hunter J, Rapkin B, Remien RH (2008). Keeping the spirit of community partnerships alive in the scale up of HIV/AIDS prevention: critical reflections on the roll out of DEBI (Diffusion of Effective Behavioral Interventions). Am J Community Psychol.

[29] Wallerstein N, Duran B (2010). Community-based participatory research contributions to intervention research: the intersection of science and practice to improve health equity. Am J Public Health.

[30] SAS 9.4 Software overview for the customer. Available from: https://support.sas.com/software/94/index.html. Cited 2021 Mar 3.

[31] Chirwa GC (2019). Socio-economic inequality in comprehensive knowledge about HIV in Malawi. Malawi Med J.

[32] Fladseth K, Gafos M, Newell ML, McGrath N (2015). The impact of gender norms on condom use among HIV-Positive adults in Kwazulu-Natal, South Africa. PLoS One.

[33] Harrison A, Colvin, CJ, Kuo C, et al. Sustained High HIV Incidence in Young Women in Southern Africa: Social, Behavioral, and Structural Factors and Emerging Intervention Approaches. Curr HIV/AIDS Rep. 2015;12:20715. 10.1007/s11904-015-0261-0. 10.1007/s11904-015-0261-0 PMC4430426 25855338

[34] Maclure R (2017). Youth reflexivity as participatory research in Senegal: a field study of reciprocal learning and incremental transformations. Socl Incl.

[35] Sommer M, Likindikoki S, Kaaya S (2015). “Bend a fish when the fish is not yet dry”: adolescent boys’ perceptions of sexual risk in Tanzania. Arch Sex Behav.

[36] AVERT. Averting HIV, HIV AIDS and AIDS in Malawi. 2018. https://www.avert.org/professionals/hiv-around-world/sub-saharan-africa/malawi. Accessed 15 Nov 2020.

[37] Rosenberg NE, Hauser BM, Ryan J, Miller WC (2016). The effect of HIV counselling and testing on HIV acquisition in sub-Saharan Africa: a systematic review. Sex Transm Infect.

[38] Yende N, Van Rie A, West NS, Bassett J, Schwartz SR (2017). Acceptability and preferences among men and women for male involvement in antenatal care. J Pregnancy.

[39] Ochillo MA, Van Teijlingen E, Hind M (2017). Influence of faith-based organisations on HIV prevention strategies in Africa: a systematic review. Afr Health Sci.

[40] National AIDS Commission Malawi. National strategic plan for HIV and AIDS. 2015–2020. Government of Malawi. 2014;(August 2011):1–81. http://www.doh.gov.za/docs/stratdocs/2012/NSPfull.pdf. Accessed 15 Nov 2021.

[41] Reaching youth through radio programs in Malawi – Population Reference Bureau. Available from: https://www.prb.org/insight/reaching-youth-through-radio-programs-in-malawi. Cited 2021 Feb 25.

[42] Chirwa G, Naidoo D (2014). Structural and social constraints in the teaching of life skills for HIV/AIDS prevention in Malawi primary schools. South Afr J Child Educ.

[43] Chirwa GW. A case study of challenges facing the implementation of life skills education in primary schools in the Zomba District, Malawi. Unpublished master’s thesis), University of Witwatersrand, South Africa. 2009.

[44] Likupe G, Chintsanya J, Magadi M, Munthali A, Makwemba M (2021). Barriers to sexual and reproductive education among in-school adolescents in Zomba and Mangochi districts. Malawi. Sex Educ.

[45] Crittenden KS, Kaponda CPN, Jere DL, McCreary LL, Norr KF (2015). Participation and diffusion effects of a peer-intervention for HIV prevention among adults in rural Malawi. Soc Sci Med.

[46] Taylor T. Ceiling effect. In: NJ Salkind, editor. Encyclopedia of research design. SAGE Publications, Inc.; 2010. pp. 133–134. 10.4135/9781412961288.n44.

[47] Aarø LE, Breivik K, Klepp KI, Kaaya S, Onya HE, Wubs A (2011). An HIV/AIDS knowledge scale for adolescents: item response theory analyses based on data from a study in South Africa and Tanzania. Health Educ Res.

[48] Ciampa PJ, Skinner SL, Patricio SR, Rothman RL, Vermund SH, Audet CM (2012). Comprehensive knowledge of HIV among women in rural Mozambique: development and validation of the HIV knowledge 27 scale. PLoS One.

[49] Jackson IL, Okonta JM, Ukwe CV. Development and psychometric evaluation of the patient’s HIV knowledge questionnaire (PHKQ). Int J Clin Pharm. 2020;42:695–702. 10.1007/s11096-020-00963-z. 10.1007/s11096-020-00963-z 31939032

