# Supplementary material for: A peer group intervention implemented by community volunteers increased HIV prevention knowledge
Source: BMC Public Health. 2023 Feb 10;23:301. doi: 10.1186/s12889-022-14715-3 (PMC9912512; doi:10.1186/s12889-022-14715-3)
Supplement: Supplementary file 1 — Additional file 1. Youth Subsample: Bivariate relationships between each covariate and knowledge measures (UNAIDS Knowledge and HIV/PMTCT Knowledge Index). Table showing bivariate relationships between all covariates and each knowledge outcome at Baseline, Survey 2 and Survey 3 for the Youth Subsample. [file 12889_2022_14715_MOESM1_ESM.docx]

| **Additional File 1. Youth Subsample: Bivariate relationships between each covariate and knowledge measures {UNAIDS Knowledge and HIV/PMTCT Knowledge Index)** | | | | | | | | | |
| --- | --- | --- | --- | --- | --- | --- | --- | --- | --- |
|  | **Baseline** | | | **Time 2** | | | **Time 3** | | |
|  | **N** | **UNAIDS**  **n (%)** | **HIV/PMTCT Knowledge Index**  **Mean (SD)** | **N** | **UNAIDS**  **n (%)** | **HIV/PMTCT Knowledge Index**  **Mean (SD)** | **N** | **UNAIDS**  **n (%)** | **HIV/PMTCT Knowledge Index**  **Mean (SD)** |
| **Overall Sample** | 548 | 213 (38.87) | 7.16 (1.73) | 504 | 234 (46.43) | 7.56 (1.65) | 493 | 273 (55.38) | 7.89 (1.45) |
| **Sex** | | | | | | | | | |
| Male | 265 | 111 (41.89) | **7.39 (1.60)**** | 239 | 120 (50.21) | **7.83 (1.44)***** | 235 | 137 (58.30) | **8.11 (1.29)**** |
| Female | 283 | 102 (36.04) | 6.94 (1.82) | 261 | 113 (43.30) | 7.33 (1.79) | 254 | 135 (53.15) | 7.73 (1.55) |
| **Education** | | | | | | | | | |
| Did not complete primary school | 295 | **94 (31.86)***** | ***6.71 (1.85) | 271 | **99 (36.53)***** | **7.09 (1.91)***** | 273 | **135 (49.45)**** | **7.59 (1.69)***** |
| Complete primary school | 196 | 90 (45.92) | 7.60 (1.48) | 182 | 108 (59.34) | 8.15 (0.98) | 176 | 111 (63.07) | 8.31 (0.91) |
| Complete secondary school | 57 | 29 (50.88) | 7.96 (1.03) | 47 | 26 (55.32) | 8.06 (1.22) | 40 | 26 (65.00) | 8.30 (0.82) |
| **Community** | | | | | | | | | |
| Community 1 | 184 | 78 (42.39) | 7.18 (2.05) | 166 | 90 (54.22) | 7.83 (1.50) | 159 | 92 (57.86) | 8.06 (1.41) |
| Community 2 | 188 | 73 (38.83) | 7.14 (1.63) | 178 | 74 (41.57) | 7.42 (1.71) | 175 | 105 (60.00) | 7.93 (1.46) |
| Community 3 | 176 | 62 (36.23) | 7.15 (1.45) | 156 | 69 (44.23) | 7.46 (1.71) | 155 | 75 (48.39) | 7.73 (1.43) |
| **Religious Involvement** | | | | | | | | | |
| Less involved | 210 | 88 (41.90) | 7.23 (1.71) | 193 | 99 (51.30) | 7.63 (1.60) | 189 | *116 (61.38) | 8.00 (1.43) |
| Very involved | 338 | 125 (36.98) | 7.11 (1.74) | 307 | 134 (43.65) | 7.53 (1.68) | 300 | 156 (52.00) | 7.85 (1.44) |
| **Education Now Baseline** |  |  |  |  |  |  |  |  |  |
| Not in School | 115 | 48 (41.74) | 7.30 (1.67) | - | - | - | - | - | - |
| In School | 433 | 165 (38.11) | 7.12 (1.74) | - | - | - | - | - | - |
| **Education Now Time 2** | | | | | | | | | |
| Not in School | - | - | - | 133 | 71 (53.38) | 7.67 (1.74) | - | - | - |
| In School | - | - | - | 371 | 163 (43.94) | 7.52 (1.62) | - | - | - |
| **Education Now Time 3** | | | | | | | | | |
| Not in School | - | - | - | - | - | - | 184 | 103 (55.98) | 7.78 (1.66) |
| In School | - | - | - | - | - | - | 308 | 169 (54.87) | 7.96 (1.32) |
| **Partner Status Baseline** | | | | | | | | | |
| Single | 474 | 186 (39.24) | 7.15 (1.76) | - | - | - | - | - | - |
| Married or living with partner | 74 | 27 (36.49) | 7.20 (1.52) | - | - | - | - | - | - |
| **Partner Status Time 2** | | | | | | | | | |
| Single | - | - | - | 441 | 200 (45.35) | 7.51 (1.70)* | - | - | - |
| Married or living with partner | - | - | - | 63 | 34 (53.97) | 7.87 (1.26) | - | - | - |
| **Partner Status Time 3** | | | | | | | | | |
| Single | - | - | - | - | - | - | 380 | 215 (56.58) | 7.94 (1.46) |
| Married or living with partner | - | - | - | - | - | - | 112 | 57 (50.89) | 7.74 (1.43) |

| **Intervention Baseline** | | | | | | | | | |
| --- | --- | --- | --- | --- | --- | --- | --- | --- | --- |
| Control | 548 | 213 (38.87) | 7.16 (1.73) | - | - | - | - | - | - |
| **Intervention Time 2** | | | | | | | | | |
| Control | - | - | - | 334 | ***143 (42.81)** | ***7.44 (1.71)** | - | - | - |
| Intervention | - | - | - | 170 | 91 (53.53) | 7.79 (1.51) | - | - | - |
| **Intervention Time 3** | | | | | | | | | |
| Control | - | - | - | - | - | - | 155 | ***75 (48.39)** | 7.73 (1.43) |
| Intervention | - | - | - | - | - | - | 338 | 198 (58.58) | 7.97 (1.46) |
| **Significance levels: * < .05, ** < .01, *** < .001** | | | | | | | | | |
