# Supplementary material for: A peer group intervention implemented by community volunteers increased HIV prevention knowledge
Source: BMC Public Health. 2023 Feb 10;23:301. doi: 10.1186/s12889-022-14715-3 (PMC9912512; doi:10.1186/s12889-022-14715-3)
Supplement: Supplementary file 2 — Additional file 2. Adult Subsample: Bivariate relationships between each covariate and knowledge measures (UNAIDS Knowledge and HIV/PMTCT Knowledge Index). Table showing bivariate relationships between all covariates and each knowledge outcome at Baseline, Survey 2 and Survey 3 for the Adult Subsample. [file 12889_2022_14715_MOESM2_ESM.docx]

| **Additional File 2. Adult Subsample: Bivariate relationships between each covariate and knowledge measures (UNAIDS Knowledge and HIV/PMTCT Knowledge Index)** | | | | | | | | | |
| --- | --- | --- | --- | --- | --- | --- | --- | --- | --- |
|  | **Baseline** | | | **Time 2** | | | **Time 3** | | |
|  | **N** | **UNAIDS**  **n (%)** | **HIV/PMTCT Knowledge Index**  **Mean (SD)** | **N** | **UNAIDS**  **n (%)** | **HIV/PMTCT Knowledge Index**  **Mean (SD)** | **N** | **UNAIDS**  **n (%)** | **HIV/PMTCT Knowledge Index**  **Mean (SD)** |
| **Overall Sample** | 460 | 216 (46.96) | 7.78 (1.36) | 426 | 238 (55.87) | 8.00 (1.26) | 434 | 249 (57.37) | 8.09 (1.24) |
| **Sex** | | | | | | | | | |
| Male | 229 | 107 (46.72) | **7.62 (1.56)**** | 206 | 114 (55.34) | **7.82 (1.37)**** | 213 | 119 (55.87) | 8.02 (1.23) |
| Female | 231 | 109 (47.19) | 7.95 (1.12) | 220 | 124 (56.36) | 8.18 (1.13) | 221 | 130 (58.82) | 8.15 (1.25) |
| **Education** | | | | | | | | | |
| Did not complete primary school | 198 | **73 (36.87)***** | **7.42 (1.63)***** | 188 | **90 (47.87)**** | 7.73 (1.48) | 192 | 105 (54.69) | **7.91 (1.32)***** |
| Complete primary school | 174 | 90 (51.72) | 8.00 (1.10) | 162 | 93 (57.41) | **8.12 (1.04)***** | 164 | 90 (54.88) | 8.07 (1.29) |
| Complete secondary school | 88 | 53 (60.23) | 8.18 (0.93) | 76 | 55 (72.37) | 8.42 (0.94) | 78 | 54 (69.23) | 8.55 (0.68) |
| **Community** | | | | | | | | | |
| Community 1 | 154 | 76 (49.35) | 7.81 (1.24) | 133 | 81 (60.90) | 8.19 (0.99) | 146 | 83 (56.85) | 8.13 (1.17) |
| Community 2 | 147 | 59 (40.14) | 7.60 (1.53) | 141 | 74 (52.48) | 7.86 (1.46) | 135 | 81 (60.00) | 8.07 (1.32) |
| Community 3 | 159 | 81 (50.94) | 7.93 (1.29) | 152 | 83 (54.61) | 7.97 (1.28) | 153 | 85 (55.56) | 8.06 (1.23) |
| **Religious Involvement** | | | | | | | | | |
| Less involved | 148 | **53** (35.81)** | **7.36 (1.73)***** | 137 | 70 (51.09) | **7.74 (1.56)**** | 140 | 74 (52.86) | **7.77 (1.58)**** |
| Very involved | 312 | 163 (52.24) | 7.98 (1.10) | 289 | 168 (58.13) | 8.13 (1.08) | 294 | 175 (59.52) | 8.24 (1.00) |
| **Partner Status Baseline** | | | | | | | | | |
| Single | 98 | 40 (40.82) | **7.50 (1.63)*** | - | - | - | - | - | - |
| Married or living with partner | 362 | 176 (48.62) | 7.86 (1.27) | - | - | - | - | - | - |
| **Partner Status Time 2** | | | | | | | | | |
| Single | - | - | - | 94 | **44 (46.81)*** | **7.69 (1.70)*** | - | - | - |
| Married or living with partner | - | - | - | 332 | 194 (58.43) | 8.09 (1.10) | - | - | - |
| **Partner Status Time 3** | | | | | | | | | |
| Single | - | - | - | - | - | - | 80 | **38 (47.50)*** | 7.78 (1.68) |
| Married or living with partner | - | - | - | - | - | - | 354 | 211 (59.60) | 8.16 (1.10) |
| **Intervention Baseline** | | | | | | | | | |
| Control | 460 | 216 (46.96) | 7.78 (1.36) | - | - | - | - | - | - |
| **Intervention Time 2** | | | | | | | | | |
| Control | - | - | - | 293 | 157 (53.58) | **7.92 (1.36)*** | - | - | - |
| Intervention | - | - | - | 133 | 81 (60.90) | 8.19 (0.99) | - | - | - |
| **Intervention Time 3** | | | | | | | | | |
| Control | - | - | - | - | - | - | 153 | 85 (55.56) | 8.06 (1.23) |
| Intervention | - | - | - | - | - | - | 281 | 164 (58.36) | 8.10 (1.24) |
| **Significance level: * < .05, ** < .01, *** < .001** | | | | | | | | | |
